# Dihydrotestosterone levels at birth associate positively with higher proportions of circulating immature/naïve CD5^+^ B cells in boys

**DOI:** 10.1038/s41598-017-15836-1

**Published:** 2017-11-14

**Authors:** Anna-Carin Lundell, Inger Nordström, Kerstin Andersson, Anna Strömbeck, Claes Ohlsson, Åsa Tivesten, Anna Rudin

**Affiliations:** 1 Department of Rheumatology and Inflammation Research, Sahlgrenska Academy, University of Gothenburg, Gothenburg, Sweden; 2 Center for Bone and Arthritis Research (CBAR), Sahlgrenska Academy, University of Gothenburg, Gothenburg, Sweden; 3 Department of Internal Medicine and Clinical Nutrition, Wallenberg Laboratory for Cardiovascular and Metabolic Research, Institute of Medicine, Sahlgrenska Academy, University of Gothenburg, Gothenburg, Sweden

## Abstract

Boys present with higher proportions of immature/naïve CD5^+^ B cells than girls up to 3 years of age. Boys also have higher fractions of regulatory T cells (Tregs) in early infancy, but the mechanisms for these sex-related differences are unknown. In the prospective FARMFLORA follow-up study of 23 boys and 25 girls, we investigated if these immunological differences remained at 8 years of age. We also examined if testosterone or dihydrotestosterone (DHT) levels at birth and at 8 years of age were associated with immune maturation. Immunological variables and androgen levels were examined and measured in blood samples obtained at birth, 3–5 days and at 8 years of age. Boys had higher proportions of CD5^+^ and immature/transitional CD24^hi^CD38^hi^ B cells, whereas girls had higher fractions of B cells with a memory phenotype at 8 years of age. School-aged boys also presented with higher frequencies of Tregs, and a greater capacity to produce T-cell-associated cytokines. Among boys, higher cord blood DHT levels were associated with higher proportions of CD5^+^ B cells in early infancy and at 8 years of life. These results suggest that DHT actions *in utero* might be involved in the mechanism for delayed peripheral B-cell maturation in boys.

## Introduction

In children, the mortality rate in less developed countries is higher among boys than girls, although they have equal access to food and medical care^1^. Moreover, boys generally mount lower antibody responses to both live attenuated and inactivated vaccines^2–5^, and also have lower total serum IgM and IgG levels than girls^4,6^. These results suggest sex-based immunological disparities, but the understanding of sex-related differences in postnatal adaptive immune maturation between boys and girls is incomplete. We have previously reported that boys present with higher proportions of circulating immature/naïve CD5^+^ B cells over the first 3 years of life^7^ as well as with higher proportions of Tregs in cord blood and in early infancy compared to girls^4^. Regulatory T cells (Tregs), in humans defined as CD4^+^CD25^+/hi^FOXP3^+^ or CD25^+^CD127^lo/neg^, play an important role in immune regulation by their ability to impede proliferation and cytokine production of other T cells, but they also have suppressive effects on B cells^8–12^. Yet, if these sex-related differences in peripheral T- and B-cell maturation persist when children reach school-age remains to be examined.

The prevalence of several autoimmune diseases, for example systemic lupus erythematosus and rheumatoid arthritis, is higher among women than men^13,14^. Therefore, a lot of emphasis have been placed on the role of female sex hormones on immune responses and on different immune cell subsets, reviewed in^15,16^. Less is known about the impact of androgens on the immune system, but androgen deficiency in men is associated with increased risk for autoimmune disease^17,18^. In male mice, castration induces expansion of both the bone marrow and the splenic B-cell population^19,20^, which can be reversed by replacement with testosterone or dihydrotestosterone (DHT)^20^. Castration of male mice also increases thymic and peripheral CD4^+^ T-cell numbers^21^. Androgens may exert their effects directly via hormone receptors expressed by lymphocytes^22–24^, but the effects of testosterone and DHT on B- and T cells may also occur indirectly by androgen targeting of stromal cells and osteoblast-lineage cells in the bone marrow^25,26^. In newborn children, umbilical cord blood testosterone and DHT levels are higher in boys compared to girls^27,28^. However, if cord blood testosterone or DHT levels are associated with peripheral adaptive immune maturation and the proportions of Tregs in early infancy and later in childhood among boys and girls has not been investigated.

To address these gaps in knowledge, we have performed a detailed immunological follow-up of the prospective FARMFLORA birth cohort study at 8 years of age. We here report that boys displayed higher proportions of immature/naïve B cells also at 8 years of age, while girls had higher fractions of B cells with a memory phenotype. Boys also presented with higher frequencies of Tregs and mononuclear cells with a greater capacity to produce cytokines. Among boys only, higher cord blood DHT, but not testosterone, levels were associated with higher proportions of immature/naïve CD5^+^ B cells in early infancy as well as at 8 years of age.

## Materials and Methods

### Subjects and collection of blood samples

In the prospective FARMFLORA study, farming and non-farming families from rural areas in the Skaraborg region in South-West Sweden were enrolled at maternity care clinics. Sixty-five healthy infants born at term (33 boys and 32 girls, median gestational age at delivery: boys 279 days and range 254–297 days, girls 279 days and range 254–298 days) were included in the study and have previously been followed in detail with respect to adaptive immune maturation up to 8 years of age^4,7,28,29^. In the 8-year follow-up study, 48 children participated (23 boys and 25 girls; median age boys: 7.9 years, range 6.8–9.1 and girls: 8.3 years, range 6.4–9.3). In this part of the study, immunological data from peripheral blood samples obtained at birth (umbilical cord), 3–5 days and 8 years of age were included. All blood samples were collected in preservative-free heparin tubes. Allergic disease at 8 years of age was clinically evaluated for all children by a pediatrician as previously described in detail^29^ and also presented in Table 1. Immunization data regarding DTP and MMR vaccine-specific antibody plasma titers from children whose parents agreed to take part in this specific part of the study are presented in previous publications^3,4^ and in Table 1. All parents provided written informed consent and the study protocol was approved by the Human Research Ethics Committee of the Medical Faculty, University of Gothenburg, Sweden. All experiments were carried out in accordance with the approved guidelines and regulations.

**Table 1:** Clinical diagnosis of allergic disease at 8 years of life, and DTP and MMR vaccine-specific antibody titers in relation to sex in the 8-year follow-up study.

	All n = 48	Boys n = 23	Girls n = 25	*P*-value^d^
Any allergy at 8 years of age^a^, n (%)	10 (21)	6 (26)	4 (16)	0.49
Eczema, n (%)	5 (10)	3 (13)	2 (8)	0.66
Asthma, n (%)	4 (8)	3 (13)	1 (4)	0.34
Allergic rhinoconjunctivitis, n (%)	3 (6)	2 (9)	1 (4)	0.60
Food allergy, n (%)	0 (0)	0 (0)	0 (0)	1.0
DTP vaccination at 3 m, boosters at 5 and 12 m, n (%)	40 (83)^b^	19 (83)	21 (84)	
IgG a-diphtheria toxin at 18 m, IU/ml (median, range)	0.67 (0.1–3.3)	0.6 (0.1–1.7)	0.8 (0.2–3.3)	0.15
IgG a-tetanus toxoid at 18 m, IU/ml (median, range)	1.8 (0.3–5.2)	2.2 (0.6–5.2)	1.5 (0.3–3.4)	0.18
IgG a-pertussis toxin at 18 m, IU/ml (median, range)	17 (3–202)	20 (6.6–123)	16 (3–202)	0.20
MMR vaccination at 18 m, n (%)	44 (92)^c^	20 (87)	24 (96)	
IgG a-measles at 36 m, mIU/ml (median, range)	2.2 (0.2–7.9)	1.9 (0.4–6.9)	3.3 (0.2–7.9)	0.26
IgG a-mumps at 36 m, titer (median, range)	447 (0–4562)	365 (0–2615)	787 (0–4562)	**0.003**
IgG a-rubella, IU/ml (median, range)	52 (14–135)	10 (16–121)	69 (14–135)	**0.006**

^a^One or more of the following diagnoses: eczema, asthma, allergic rhinoconjunctivitis or food allergy.

^b^Missing data from 8 children whose parents did not want to participate in this part of the study.

^c^Missing data from 4 children whose parents did not want to participate in this part of the study.

^d^Statistical difference between boys and girls (Fisher’s exact test or Mann-Whitney U-test).

Data regarding allergic disease at 8 years of age have been published previously by Strömbeck *et al*. (number 29 in the reference list), and some of the data regarding DTP and MMR vaccine-specific antibody titers have been published previously by Strömbeck *et al*. (number 3 and 4 in the reference list).

### Flow cytometry

Phenotypic characterization of lymphocytes was performed on whole blood. Immature/naïve and memory B cells were identified by CD5 and CD27 expression, respectively. Naïve and memory CD4^+^ T cells were identified by CD45RA and CD45RO expression, respectively. To identify putative Tregs, CD25 in combination with FOXP3 or CTLA-4 was used. At 8 years of age additional markers were included into the flow cytometry panel: CD24 and CD38 were added to distinguish between immature/transitional (CD24^hi^CD38^hi^), mature/naïve (CD24^int^CD38^int^), memory (CD24^hi^CD38^lo/neg^) B cells, and CD127 was added to identify CD4^+^CD25^+^CD127^lo/neg^ putative Tregs. All anti-human monoclonal antibodies are listed in Supplementary Table S1. The FOXP3 Staining Buffer Kit was purchased from eBioscience (San Diego, USA). For detection of CTLA-4 the Cytofix/Cytoperm Fixation/Permeabilization Solution Kit was used (BD Biosciences, Erembodegem Belgium). To assess total B- and T-cell numbers, TruCOUNT Tubes were used (BD Biosciences). Cord and 3–5 days blood samples were run in a FACS-Calibur (BD Biosciences) equipped with CellQuestPro software. All 8 years samples were run in a FACSCanto II (BDBiosciences) equipped with FACSDiva software. The samples were analysed with FlowJo software (TreeStar, Ashland, Oregon, USA).

### Quantification of immunoglobulins by ELISA

Plasma was prepared from collected venous blood samples and stored at −80 °C. Total IgM, IgG and IgA levels were determined by ELISA by coating plates (Nunc, Roskilde, Denmark) with goat anti-human IgM, IgG or IgA (Jackson, ImmunoResearch, Suffolk, United Kingdom), after which diluted plasma and standards were added (polyclonal IgM, IgG or IgA from human plasma; Calbiochem, Darmstadt, Germany). Detection was performed with horseradish peroxidase-conjugated goat anti-human IgM, IgG or IgA (Jackson Immunoresearch), followed by O-fenylenediamine dihydrochloride (Sigma-Aldrich, St Lois, USA) and addition of H_2_O_2_ as substrate. To measure total IgE levels, ImmunoCAP® (Phadia AB, Uppsala, Sweden) was used.

### Androgen measurement by gas chromatography-tandem mass spectrometry

Measurement of androgens in plasma, including testosterone and dihydrotestosterone (DHT), were measured in a single run by gas chromatography-tandem mass spectrometry (GC-MS/MS) as described previously^28,30^. In summary, after the addition of isotype-labeled standards, steroids were extracted with chlorobutane and purified on a silica column. Next, they were derivatized using pentafluorobenzylhydroxylamine hydrochloride followed by pentafluorobenzoyl chloride. Steroids were analyzed using an Agilent 7000 triple quadrupole mass spectrometer equipped with a chemical ionization source. This was done in multiple reactions monitoring mode with ammonia as reagent gas. Lower limit of detection (LOD) for the assay is 4 and 1.6 pg/ml and lower limit of quantification (LOQ) 8 and 2.5 pg/ml for testosterone and DHT, respectively^30^. At birth, 4 female plasma samples were below the LOQ for DHT; these were assigned double LOQ (i.e. 5 pg/ml) due to dilution of samples (1:2). At 8 years of age, 5 female and 4 male undiluted samples were below the LOQ for DHT and 2 female samples for testosterone; these were assigned LOQ (2.5 pg/ml and 8 pg/ml, respectively).

### Isolation of peripheral blood mononuclear cells

Peripheral blood mononuclear cells (PBMC) from blood samples of the 8-year-old children were isolated by density gradient centrifugation on Lymphoprep^TM^ (Axis-Shield PoC AS, Oslo, Norway). Interface MNC were collected, washed three times with phosphate buffer (PBS) (HyClone^TM^, Logan, USA) and resuspended in serum free AIM-V medium with gentamycin (Gibco, Thermo Fischer Scientific, Waltham, USA) supplemented with 40 µM mercaptoethanol (Merck Millipore, Darmstadt, Germany). The MNC were cultured at 10^6^ cells/ml in 96-well flat-bottomed plates with medium alone or with 5 µg/ml phytohaemagglutinin (PHA) (Roche Diagnostics GmbH, Mannheim, Germany). The cells were incubated at 5% CO_2_ for 24 hours. The supernatants were collected and stored at −20 °C until cytokine levels were determined.

### Quantification of cytokines by flow cytometry

To analyze the supernatants, CBA Flex Set (BDBiosciences) was used according to the supplier’s instructions. The cytokines examined were IFN-γ, IL-4, IL-5, IL-13 and IL-17A. Samples were acquired in a FACSVerse equipped with FACSuite software and analyzed with FCAP Array software.

### Statistical analysis

Multivariate OPLS with discriminant analysis (DA) was used to obtain a maximum separation of X-variables, i.e. B- and T-cell maturation variables, based on class information, e.g. girl (yes/no). OPLS analysis was implemented to investigate associations between a selected Y-variable and X-variables (SIMCA 13.0; Umetrics, Umeå, Sweden). The scale presented on the Y-axis of the OPLS plot is a dimensionless scale, the loading vector is normalized to length one. The quality of OPLS analyses is based on R2, how well the variation of the variables is explained by the model, and Q2, how well a variable can be predicted. The final OPLS plots in Figs 3–5 are models based on X-variables with variable influence of projection (VIP values) >0.5. VIP values can be used to discriminate between more or less important predictors for the overall model. To avoid biased preselection and mass significance, non-parametric two-tailed Mann-Whitney U-test and Spearman’s rank test (GraphPad Software; La Jolla, USA) were performed exclusively on the X-variables that contributed most to the respective multivariate model. A *P*-value of 0.05 or less was regarded as statistically significant. Significant differences are indicated with asterisks (added in retrospect) in the OPLS-plots. Multiple linear regression was used to estimate the relationship between the proportion of CD5^+^ B cells at different ages (dependent variable) and DHT levels at birth, gestational age at delivery, BAFF levels at birth and vaccine-specific antibody titers (explanatory variables) (SPSS version 24.0 for Windows, Inc., Chicago, USA). The D’Agostino and Pearson omnibus normality test was used to assess if the data were normally distributed (GraphPad).

## Results

### School-aged boys have higher proportions of circulating immature/naive B cells than girls

**Figure 1:**
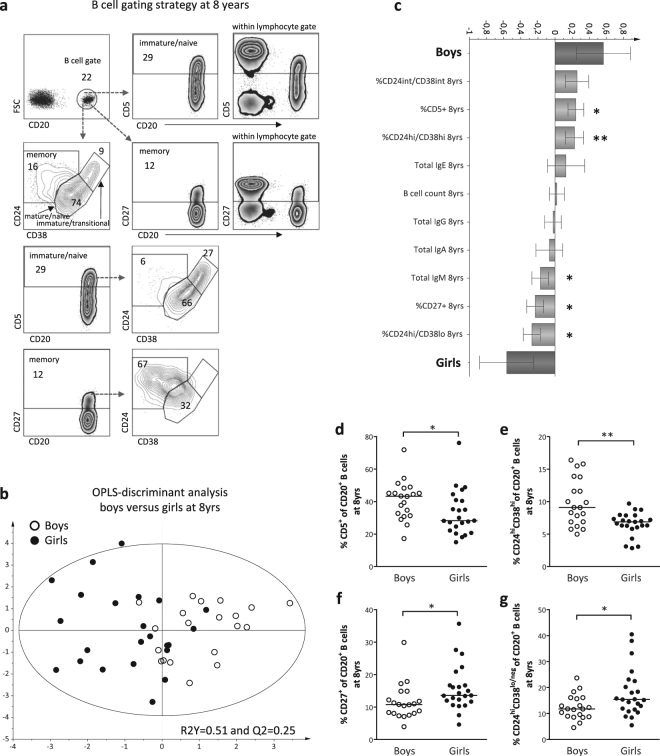
Boys have higher proportions of immature/naïve B cells at 8 years. (**a**) B-cell gating strategy at 8 years of age. (**b**) OPLS-DA plot displaying a separation between boys and girls and (**c**) OPLS loading column plot depicting sex-related associations with respect to the B-cell variables assessed. (**d**) Proportions of CD5^+^ of CD20^+^ B cells, (**e**) proportions of transitional (CD24^hi^CD38^hi^) of CD20^+^ B cells, (**f**) proportions of CD27^+^ of CD20^+^ B cells and (**g**) proportions of CD24^hi^CD38^lo/neg^ of CD20^+^ B cells in boys and girls at 8 years of age. *P ≤ 0.05, **P ≤ 0.01 (Mann-Whitney U-test).

We have previously demonstrated that boys have higher proportions of immature/naïve CD5^+^ B cells in blood than girls over the first three years of life^7^. By multivariate factor analysis, we investigated whether B-cell maturation differed between sexes also at 8 years of age. The B-cell gating strategy is presented in Fig. 1a, which also shows that the vast majority of the CD5^+^ B cells reside within the CD24^hi/int^CD38^hi/int^ immature/naïve gates, while the majority of CD27^+^ B cells are found in the CD24^hi^CD38^lo^ memory gate. OPLS-discriminant analysis demonstrated a separation between boys and girls based on the B-cell maturation variables assessed (Fig. 1b). Male sex was associated with higher proportions of CD5^+^ B cells and CD24^hi^CD38^hi^ immature (transitional) B cells (Fig. 1c), which was corroborated by univariate analysis demonstrating that boys had significantly higher proportions of these two cell subsets compared to girls at 8 years of age (Fig. 1d and e). Female sex associated with higher proportions of CD27^+^ and CD24^hi^CD38^lo^ memory B cells (Fig. 1c), which were also significantly higher among girls in univariate analysis (Fig. 1f and g). Additionally, girls had higher levels of circulating total IgM compared to boys (Fig. 1c). Current allergic disease could be a potential confounding factor for these findings, but comparable results were obtained when children with allergic disease at 8 years of age were excluded from the analyses (Supplementary Fig. S1). As boys present with lower BAFF levels in cord blood and respond with lower vaccine-induced anti-mumps and anti-rubella titers compared to girls^4,7^, we also examined if these factors were related to the proportions of immature/naïve B cells in non-allergic children at 8 years of life. In multiple regression analysis, only male sex contributed independently to higher proportions of transitional CD24^hi^CD38^hi^ B cells (*P* = 0.02). For CD5^+^ B cells, however, none of the variables examined contributed independently to higher proportions of these cells. In summary, these results demonstrate that boys have higher proportions of circulating immature/naïve B cells and lower proportions of memory B cells than girls at school age, and that this was unrelated to presence of allergic disease, vaccine-specific anti-mumps or anti-rubella response or BAFF levels at birth.

### School-aged boys have higher proportions of circulating Tregs and produce higher T-cell-related cytokine levels than girls

**Figure 2:**
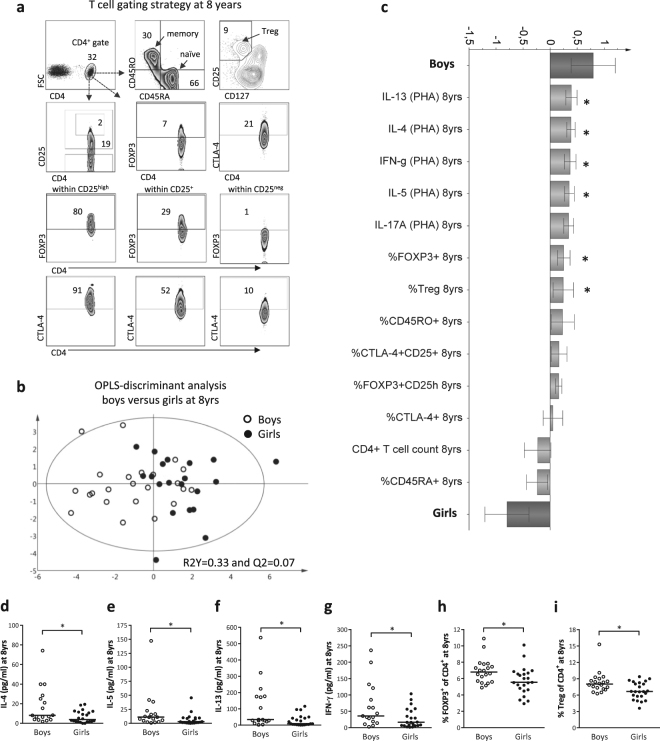
Boys and girls differ in T-cell maturation at 8 years of age. (**a**) T-cell gating strategy at 8 years of age. (**b**) OPLS-DA plot displaying a separation between boys and girls and (**c**) OPLS loading column plot depicting sex-related associations with respect to the T-cell variables assessed. (**d**) PHA-induced concentrations of IL-4, (**e**) IL-5, (**f**) IL-13 and (**g**) IFN-γ produced by PBMCs from boys and girls at 8 years of age. (**h**) Proportions of FOXP3^+^ of CD4^+^ T cells and (**i**) proportions of Treg of CD4^+^ T cells in boys and girls at 8 years of age. *P ≤ 0.05 (Mann-Whitney U-test).

Boys have higher fractions of putative Tregs in blood compared to girls in early infancy^4^. Therefore we next investigated whether T-cell maturation differed between sexes also at 8 years of age. We assessed the proportions of putative Tregs, naïve and memory CD4^+^ T cells, as well as PHA-induced T-cell-associated cytokine production. The T-cell gating strategy is presented in Fig. 2a. OPLS-discriminant analysis showed a separation between boys and girls also for T-cell variables (Fig. 2b). Male sex associated positively with T-cell-related cytokine production induced by PHA stimulation (Fig. 2c). In univariate analysis, levels of IL-4, IL-5, IL-13 and IFN-γ were higher in the supernatants after PHA-stimulation of PBMCs from boys relative to girls (Fig. 2d–g). Furthermore, the proportions of FOXP3^+^ and Treg (CD25^+^CD127^lo/neg^) within the CD4^+^ T-cell population also associated positively with male sex (Fig. 2c), and were significantly higher in boys (Fig. 2h and i). Current allergic disease was not a confounding factor as similar results were obtained when allergic children were excluded from the analyses (Supplementary Fig. S2). Taken together, boys have higher proportions of putative Tregs than girls not only in early infancy but also at school age. Male sex was also associated with increased maturation of effector T cells at 8 years of age, as measured by both Th1 and Th2 cytokine-producing capacity.

### Cord blood DHT levels are associated with higher fractions of immature/naïve B cells among boys

In the FARMFLORA study, we recently reported sex-based differences in human umbilical cord blood androgen levels; DHT levels were 2.4-fold higher in boys compared to girls, while the difference was less pronounced for testosterone (1.3-fold higher)^28^ (Supplementary Fig. 3a and c). At 8 years of age, DHT and testosterone levels did not differ between sexes (Supplementary Fig. 3b and d). Also, the concentrations of DHT at 8 years of age were lower compared to those measured in cord blood; median DHT levels in boys decreased 8-fold from birth to 8 years of age (median at birth 27.8 pg/ml, range 10.9–90.3 pg/ml vs median at 8 years of age 3.5 pg/ml, range 2.5–25.8 pg/ml, *P* = <0.0001) and 2-fold in girls (median at birth 11.5 pg/ml, range 5–32.7 pg/ml vs median at 8 years of age 5.3 pg/ml, range 2.5–30.3 pg/ml, *P* = 0.01). The levels of testosterone decreased drastically from birth to 8 years of age in both sexes (Supplementary Fig. 3c-d). Also, DHT or testosterone levels at birth did not correlate to those measured at 8 years of age in either boys or girls (Supplementary Fig. 3e–h).

**Figure 3:**
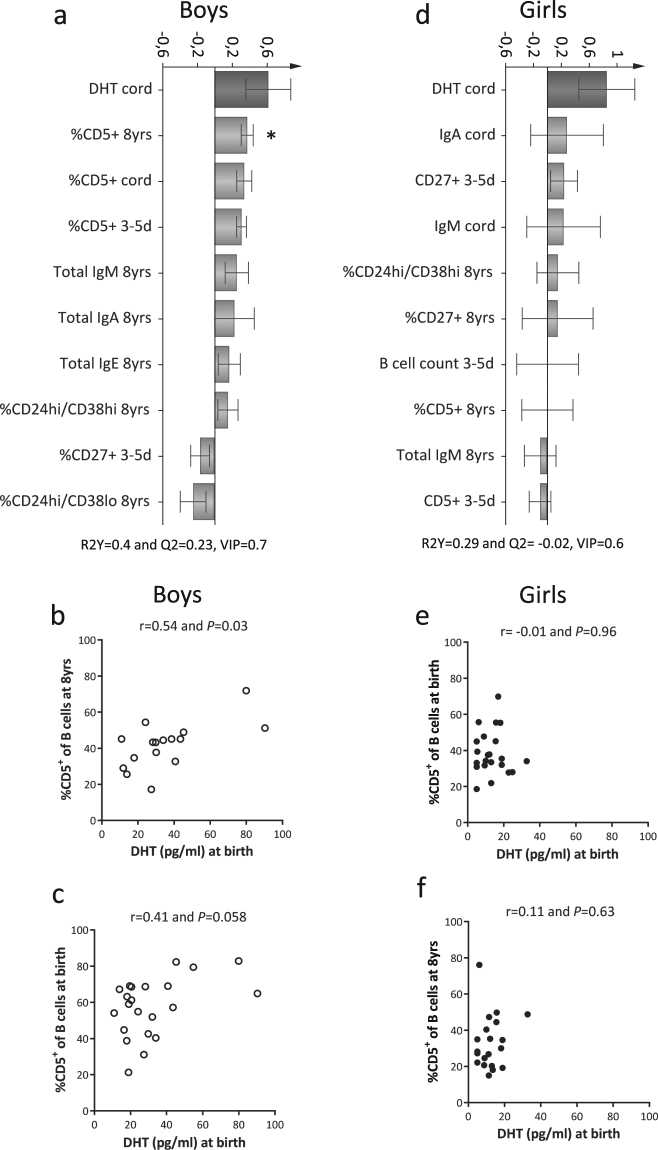
Cord blood DHT levels are associated with higher proportions of CD5^+^ B cells in boys. (**a** and **d**) OPLS column loading plots displaying association between cord blood DHT levels and B-cell variables in boys and in girls, respectively. (**b** and **e**) Correlations between cord blood DHT levels and proportions of CD5^+^ B cells at 8 years of age and (**c** and **f**) at birth in boys and girls, respectively. *P ≤ 0.05 (Spearman’s rank correlation test).

The differences in blood levels of DHT and testosterone at birth between boys and girls prompted us to investigate the associations between levels of these androgens and postnatal B- and T-cell maturation. Cord blood DHT levels in boys were associated with higher proportions of CD5^+^ B cells at birth, 3–5 days and also at 8 years of age in multivariate analysis (Fig. 3a). In non-parametric Spearman rank correlation analysis, there was a significant correlation between DHT levels and CD5^+^ B cells at 8 years of age (Fig. 3b) and a trend for correlation between DHT levels and proportions of CD5^+^ B cells at birth (Fig. 3c). Gestational age at delivery could be a potential confounding factor for these results as it is inversely correlated with DHT levels at birth in boys^28^. However, in multiple linear regression analysis, log-transformed normally distributed DHT levels contributed independently to higher proportions of CD5^+^ B cells at 8 years of age (*P* = 0.02). Gestational age at delivery exerted an independent effect on the proportion of CD5^+^ B cells at 3–5 days of age (*P* = 0.001). In girls, DHT levels were unrelated to B-cell variables assessed (Fig. 3d–f). DHT levels at 8 years of age were unrelated to B-cell maturation in both sexes (Supplementary Fig. 4a,b).

**Figure 4:**
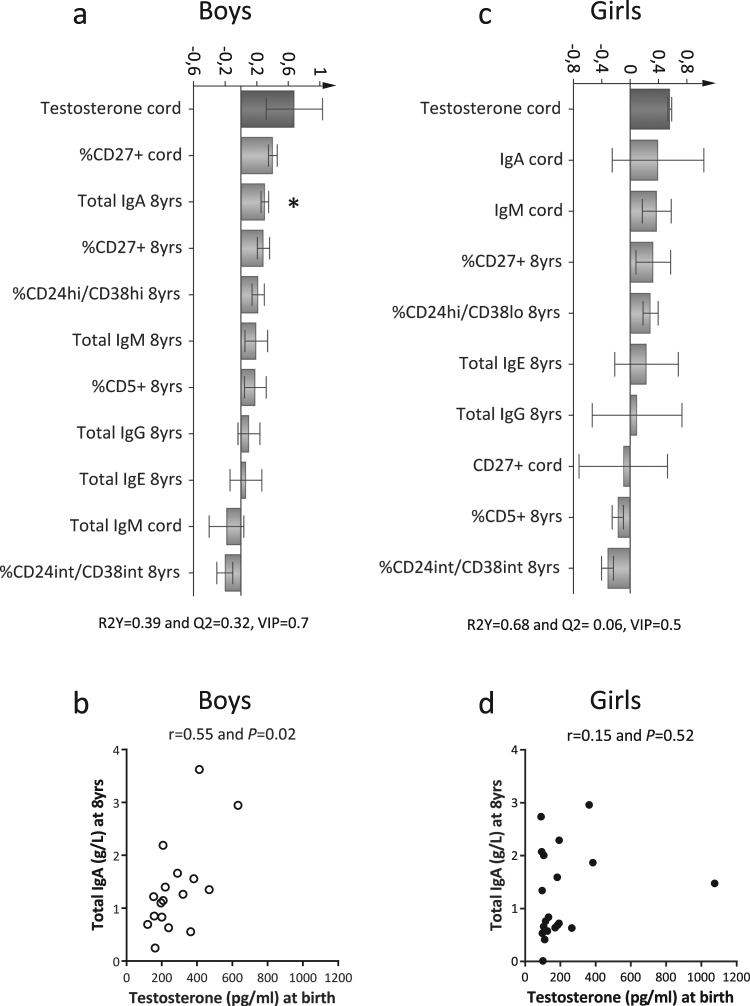
Cord blood testosterone levels are associated with B-cell maturation/activation in boys. (**a** and **c**) OPLS column loading plots demonstrating associations between cord blood testosterone levels and B-cell variables in boys and in girls, respectively. (**b** and **d**) Correlations between cord blood testosterone levels and total IgA levels at 8 years of age in boys and in girls, respectively. *P ≤ 0.05 (Spearman’s rank correlation test).

Cord blood testosterone levels in boys were associated with higher proportions of CD27^+^ memory B cells, both in infancy and at school age, and with higher IgA levels at 8 years of age (Fig. 4a), albeit only for the latter, there was a significant correlation (Fig. 4b). In girls, testosterone levels were unrelated to B-cell maturation (Fig. 4c,d). Testosterone levels at 8 years of age were unrelated to B-cell maturation in both sexes (Supplementary Fig. 5a,b).

**Figure 5:**
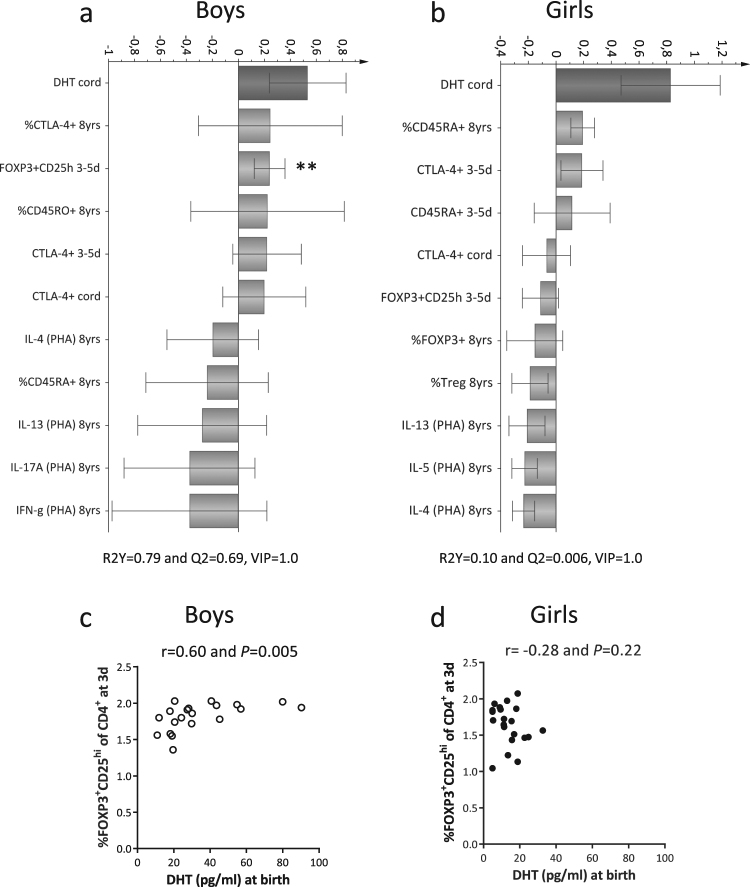
Cord blood DHT levels are associated with higher proportions of Tregs in early infancy among boys. (**a,b**) OPLS column plots demonstrating association between cord blood DHT levels and T-cell variables in boys and in girls, respectively. (**c,d**) Correlations between cord blood DHT levels and proportions of FOXP3^+^CD25^hi^ of CD4^+^ at 3 days of age in boys and in girls, respectively. **P ≤ 0.01 (Spearman’s rank correlation test).

Regarding T-cell maturation and androgen levels, DHT levels in boys were related to higher proportions of memory CD45RO^+^ cells, and T cells with a regulatory phenotype, i.e. CTLA-4^+^ or FOXP3^+^CD25^hi^ within the CD4^+^ T-cell population at 3–5 days of age (Fig. 5a). In Spearman rank correlation analysis, cord blood DHT levels correlated with the fractions of putative Tregs (FOXP3^+^CD25^hi^) at 3–5 days in boys, but not in girls (Fig. 5c,d). In multivariate regression analysis however, neither log-transformed DHT levels nor gestational age at delivery contributed to the proportion of putative Tregs at 3–5 days of age (*P* = 0.12 and *P* = 0.29, respectively). Levels of DHT at 8 years (Supplementary Fig. 4c-d) and levels of testosterone at birth and at 8 years of age were unrelated to postnatal T-cell maturation in both sexes (Supplementary Fig. 5c,d). In summary, higher cord blood DHT levels were associated with increased proportions of circulating immature/naïve B cells in 8-year-old boys independently of gestational age at delivery.

## Discussion

There are sex-related differences in proportions of certain B- and T-cell subsets in blood as well as in adaptive immune responses, but the underlying mechanisms for these differences remains to be illuminated. We here report that school-aged boys have higher proportions of circulating CD5^+^ B cells, which includes both immature/transitional and mature/naïve cells, as well as higher fractions of Treg compared to girls at the same age. Another novel finding was that DHT but not testosterone levels at birth were positively associated with higher proportions of CD5^+^ B cells in early infancy and at 8 years of age. Thus, DHT might be involved in the mechanism that explains delayed peripheral B-cell maturation in male sex during childhood.

Boys display strikingly higher proportions of immature/naïve CD5^+^ B cells in blood already at birth and over the following 3 years of life relative to girls^7^. From the same prospective birth cohort study, we here show that boys had higher proportions of CD5^+^ B cells compared to girls also when the children had reached school-age. At school age, boys also displayed lower proportions of B cells with a memory phenotype relative to girls. Finally, total IgM levels were lower among boys than girls, which is in accordance with several previous observations^4,6,31,32^. We have previously shown that a high proportion of CD5^+^ B cells in the first month of life is a risk factor for allergy development and that there is a higher prevalence of allergic disease among boys compared to girls in the first years of life^33^. When children with allergic disease at 8 years of age were excluded from the analyses in the present study boys still had significantly higher proportions of immature/naïve B cells relative to girls, which demonstrates that current allergy is not a confounding factor for sex-related differences in B cell maturation in school-aged children. We have also reported that boys present with lower circulating BAFF levels at birth and that they respond with lower vaccine-induced anti-mumps and anti-rubella antibody titers compared to girls^3,4^. In multiple regression analysis, only male sex contributed independently to higher proportions of immature B cells at 8 years of age. Immature/naïve CD5^+^ B cells per se may not necessarily be involved in immunological mechanisms that lead to lower antibody titers in boys. Instead, a high proportion of immature/naïve CD5^+^ B cells might be a biomarker that reflects a less mature and activated humoral immune system in general among boys, which in turn could influence the magnitude of both infection- and vaccine-induced antibody responses.

Male sex thus appears to be an important factor related to higher proportions of circulating CD5^+^ B cells during childhood. The fact that newborn boys have higher proportions of CD5^+^ B cells as well as higher concentrations of DHT and testosterone in blood compared to girls^28^, prompted us to investigate androgen levels in relation to postnatal adaptive immune maturation. For the first time, we here report that DHT but not testosterone levels at birth were related to higher proportions of CD5^+^ B cells in cord blood, 3–5 days and also at 8 years of age among boys. In humans, B-cell development begins early in fetal life. B cells are detected in the liver at 8 weeks and then appear in the bone marrow from approximately the 12^th^ week of gestation, and from mid-gestation and onward, the bone marrow is the major site of B-cell generation^34–36^. Interestingly, the human liver as well as the bone marrow are androgen responsive tissues as they express the androgen receptor^37–39^. Even though androgen concentrations in cord blood only reflect the androgen milieu at birth and not during gestation, sex-related differences in the hormonal milieu may emerge already *in utero* and thereby affect B-cell development in a sex-dependent manner, possibly via liver and bone marrow stromal cells. How hormonal influences during the prenatal period could have long lasting or permanent effects on postnatal immune development in humans has not been examined and very little is known from animal models. However, the term “immunological imprinting” was introduced nearly 30 years ago and refers to permanent changes induced in the immune system as a consequence of exposure to sex hormones *in utero* ^40,41^. Similarly, anatomical, physiological and behavioral sex-related traits as adults caused by intrauterine hormone exposure, i.e. “neurological imprinting”, have also been reported^42^. Thus, higher DHT levels in male newborns could be involved in the mechanism for delayed peripheral B-cell maturation during childhood, which may be imprinted already in the male fetus. Reversible effects of androgens on B-cell development have on the other hand clearly been described in animal models^43^. Removal of endogenously produced androgens by castration of male mice induces expansion of both the bone marrow and the splenic B-cell populations, which can be reversed by androgen treatment^20^. The suppressive androgenic effect on B lymphopoiesis has been shown to be mediated via androgen-sensitive stromal cells in the bone marrow^20,25,26,44^.

In the present cohort, testosterone levels at birth were also higher in boys compared to girls but did not display the same multivariate association pattern with B-cell maturation as that of DHT. The reason for these differences can only be speculated upon, but DHT, which is converted by 5α-reductase from testosterone, is a more potent agonist to the androgen receptor than testosterone^45^. Moreover, a “non-classical” pathway leading to DHT synthesis from progesterone that does not pass the testosterone step has been described, and this pathway is particularly active in the fetus and early infancy in male sex^46,47^. Finally, testosterone, but not DHT, can be converted to estradiol by aromatase, which thereby diversifies its androgen action by activating estrogen receptors^48^.

Sex-related differences have been demonstrated for the distribution of certain T-cell subsets. For example, male infants and adults display higher fractions of Treg in blood compared to females, as shown by our group and others^4,49,50^. In the present study we demonstrate that also school-aged boys had significantly higher fractions of these cells in blood relative to girls. Moreover, DHT levels at birth were associated with higher fractions of Tregs at 3–5 days of age in boys in multivariate and correlation analysis. However, in multiple regression analysis, neither DHT levels at birth nor gestational age at delivery exerted an independent effect on the proportion of Treg at 3–5 days in boys. Regarding B-cell maturation, DHT levels at birth but not gestational age contributed to independently to higher proportions of immature/naïve B cells in boys. The significance of higher fractions of Treg in the circulation in male sex remains to be investigated further, but several conditions illustrate the importance of functioning Tregs to human immune homeostasis. For example, *Foxp3* deficiency results in IPEX syndrome that is characterized by expression of multiple autoimmune disorders^51,52^.

A strength of this study is that it is prospective and that we had the unique opportunity to measure and analyze detailed immunological data over the first 8 years of life as well as androgen data from the same children. Another advantage is that we have used multivariate analysis, which enables discovery of patterns and trends and preclude biased preselection of variables for univariate analysis. We have also used state-of-the-art technology to measure androgen concentrations, i.e. gas chromatography-tandem mass spectrometry. A limitation is the relatively small study sample, which may be reflected in the lack of statistical significance in some of the analyses. Also, outliers may have a greater impact on statistical analyses with a relatively small study sample. However, inter-individual variations and outliers are common features when investigating immunological factors in human subjects. Therefore we never exclude outliers in our data sets, but instead endeavor to present all raw data.

In conclusion, the present results suggest that DHT *in utero* might be involved in the mechanism that leads to higher proportions of circulating immature/naïve B cells and higher fractions of Tregs in boys compared to girls. We have previously demonstrated that a high proportion of CD5^+^ B cells in the first month of life is a risk factor for development of IgE-mediated allergic disease up to 8 years of age^29,33^, but it remains to be investigated if sex-related differences concerning steroid hormones and certain immune variables in early life influence development of autoimmune and infectious disease later in life.

## Electronic supplementary material

Supplementary dataset
