# Supplementary material for: Dihydrotestosterone levels at birth associate positively with higher proportions of circulating immature/naïve CD5+ B cells in boys
Source: Sci Rep. 2017 Nov 14;7:15503. doi: 10.1038/s41598-017-15836-1 (PMC5686210; doi:10.1038/s41598-017-15836-1)
Supplement: Supplementary file 1 — Supplementary dataset [file 41598_2017_15836_MOESM1_ESM.doc]

**Dihydrotestosterone levels at birth associate positively with higher proportions of circulating immature/naïve CD5^+^ B cells in boys**

Anna-Carin Lundell^1*^, Inger Nordström^1^, Kerstin Andersson^1^, Anna Strömbeck^1^, Claes Ohlsson^2^, Åsa Tivesten^3^ and Anna Rudin^1^

^1^Department of Rheumatology and Inflammation Research, ^2^Center for Bone and Arthritis Research (CBAR) and ^3^Wallenberg Laboratory for Cardiovascular and Metabolic Research, Department of Internal Medicine and Clinical Nutrition, Institute of Medicine, Sahlgrenska Academy, University of Gothenburg, Sweden

**Supplementary Table S1.** Anti-human monoclonal antibodies used for flow cytometry

| **Reactivity** | **Fluorochrome** | **Clone** | **Company** |
| --- | --- | --- | --- |
| CD4 (0-5days) | PerCP | SK3 | BD Biosciences |
| CD4 (8yrs) | APC-H7 | SK3 | BD Biosciences |
| CD25 (0-8yrs) | APC/BV421 | 2A3/BC96 | BD Biosciences/Biolegend |
| CD127 (8yrs) | FITC | HIL-7R-M21 | BD Biosciences |
| CD127 (8yrs) | Alexa Fluor 647 | HIL-7R-M21 | BD Biosciences |
| CD45RO (0-8yrs) | PE | UCHL-1 | BD Biosciences |
| FOXP3 (0-8yrs) | PE | PCH101 | eBioscience |
| Biotin CTLA-4 (0-8yrs) | PE-streptavidin | BNI3 | BD Biosciences |
| CD20 (0-5days) | PerCP | L27 | BD Biosciences |
| CD20 (8yrs) | APC-H7 | L27 | BD Biosciences |
| CD24 (8yrs) | Alexa Fluor 647 | ML5 | BD Biosciences |
| CD38 (8yrs) | PE | HB7 | BD Biosciences |
| CD5 (0-5days) | APC | UCHT2 | BD Biosciences |
| CD5 (8yrs) | Brilliant Violet 421 | UCHT2 | Biolegend |
| CD27 (0-8yrs) | FITC | L128 | BD Biosciences |

**Supplementary Figure S1**

a

b

c

d

e

**

*

*

**Boys**

**Girls**

*

**Figure S1: Proportions of B-cell subsets and total immunoglobulin levels in boys and girls without current allergic disease at 8 years of age. (a)** OPLS-DA loading column plot depicting sex-related associations regarding B-cell variables assessed in non-allergic children at 8 years of age. **(b)** Proportions of CD5^+^ of CD20^+^ B cells, **(c)** proportions of immature transitional (CD24^hi^CD38^hi^) of CD20^+^ B cells, **(d)** proportions of CD27^+^ of CD20^+^ B cells and **(e)** proportions of CD24^hi^CD38^lo/neg^ of CD20^+^ B cells in non-allergic boys and girls at 8 years of age. **P*≤0.05, ***P*≤0.01 (Mann-Whitney U-test).

**Supplementary Figure S2**

a

b

c

d

e

f

g

**Boys**

**Girls**

*

*

*

**Figure S2: Proportions of T-cell subsets and PHA-induced cytokine secretion in boys and girls without current allergic disease at 8 years of age. (a)** OPLS-DA loading column plot depicting

sex-related associations regarding T-cell variables assessed in non-allergic children at 8 years of age. **(b)** PHA-induced concentrations of IL-4, **(c)** IL-5, **(d)** IL-13 and **(e)** IFN-γ produced by PBMCs from non-allergic boys and girls at 8 years of age. **(f)** Proportions of FOXP3^+^ of CD4^+^ T cells and **(g)** proportions of Treg (CD4^+^CD25^+^CD127^lo/neg^) of CD4^+^ T cells in boys and girls at 8 years of age. **P*≤0.05 (Mann-Whitney U-test).

**Supplementary Figure S3**

a

b

c

d

At birth

At 8 yrs

At birth

At 8 yrs

e

f

g

h

**Figure S3: Levels of androgens in umbilical cord blood and at 8 years of age according to sex.**

**(a-b)** Dihydrotestosterone (DHT) levels and **(c-d)** testosterone levels in boys versus girls at birth and at 8 years of age, respectively. *****P*≤0.0001, ***P*≤0.01 (Mann-Whitney U-test). **(e and g)** Correlations between DHT or testosterone levels at birth and at 8 years of age in boys. **(f and h)** Correlations between DHT or testosterone levels at birth and at 8 years of age in girls. Mann-Whitney U-test (a-d) and Spearman’s rank correlation test (e-h). All data regarding androgen levels in cord blood have been published previously by Lundell et al (number 28 in the reference list).

**Supplementary Figure S4**

R2Y=0.46 and Q2= -0.09

Boys

a

Girls

b

R2Y=0.15 and Q2= -0.14

R2Y=0.16 and Q2= -0.53

R2Y=0.21 and Q2= -0.30

c

d

Boys

Girls

**Figure S4: Levels of DHT at 8 years in relation to B- and T-cell maturation at the same age in boys and girls.** DHT levels at 8 years of age in relation to **(a and b)** B-cell or **(c and d)** T-cell maturation variables at the same age in boys and girls, respectively.

**Supplementary Figure S5**

R2Y=0.59 and Q2= -0.33

R2Y=0.32 and Q2= -0.20

Boys

a

Girls

b

R2Y=0.35 and Q2= -0.38

R2Y=0.14 and Q2= -0.19

Boys

c

Girls

d

**Figure S5**: **Levels of testosterone at 8 years in relation to B- and T-cell maturation at the same age in boys and girls.** Testosterone levels at 8 years of age in relation to **(a and b)** B-cell or **(c and d)** T-cell maturation variables at the same age in boys and girls, respectively.
